# Trophic response to ecological conditions of habitats: Evidence from trophic variability of freshwater fish

**DOI:** 10.1002/ece3.6451

**Published:** 2020-07-03

**Authors:** Bohyung Choi, Changhwa Lee, Yuko Takizawa, Yoshito Chikaraishi, Hye‐Ji Oh, Kwang‐Hyeon Chang, Min‐Ho Jang, Hyun‐Woo Kim, Kyung‐Lak Lee, Kyung‐Hoon Shin

**Affiliations:** ^1^ Department of Marine Sciences and Convergent Technology Hanyang University Ansan Korea; ^2^ Institute of Low Temperature Science Hokkaido University Sapporo Japan; ^3^ Japan Agency for Marine‐Earth Science and Technology Yokosuka Japan; ^4^ Department of Environmental Science and Engineering Kyung Hee University Yongin Korea; ^5^ Department of Biology Education Kongju National University Gongju Korea; ^6^ Department of Environmental Education Sunchon National University Sunchon Korea; ^7^ Watershed Ecology Research Team National Institute of Environmental Research Incheon Korea

**Keywords:** CSIA of amino acids, generalists, nitrogen stable isotope, specialists, trophic niche, trophic position

## Abstract

1. To adapt to ecological and environmental conditions, species can change their ecological niche (e.g., interactions among species) and function (e.g., prey‐predation, diet competition, and habitat segregation) at the species and guild levels. Stable isotope analysis of bulk carbon and nitrogen of organisms has conventionally been used to evaluate such adaptabilities in the scenopoetic and bionomic views as the isotopic niche width.
2. Compound‐specific stable isotope analysis (CSIA) of nitrogen within amino acids provides trophic information without any disruption of scenopoetic views in the isotope ratios, unlike conventional bulk isotope analysis provides both information and therefore frequently hinders its usefulness for trophic information.
3. We performed CSIA of amino acids to understand the trophic variability of the pike gudgeon *Pseudogobio esocinus* and largemouth bass *Micropterus salmoides* as representative specialist and generalist fish species, respectively, from 16 ecologically variable habitats in the four major rivers of Korea.
4. There was little variation (1*σ*) in the trophic position (TP) among habitats for *P. esocinus* (± 0.2); however, there was considerably large variation for *M. salmoides* (± 0.6). The TP of *M. salmoides* was negatively correlated with the benthic invertebrate indices of the habitats, whereas the TP of *P. esocinus* showed no significant correlation with any indices. Thus, these two representative fish species have different trophic responses to ecological conditions, which is related to known differences in the trophic niche between specialists (i.e., small niche width) and generalists (i.e., large niche width).
5. Over the past four decades, the conventional bulk isotope analysis has not been capable of deconvoluting “scenopoetic” and “bionomic” information. However, in the present study, we demonstrated that the CSIA of amino acids could isolate trophic niches from the traditional ecological niche composed of trophic and habitat information and evaluated how biological and ecological indices influence the trophic response of specialists and generalists.

## Graphical abstract

We first applied compound‐specific stable isotope analysis of amino acids to the ecological niche studies, in order to isolate trophic niche that is accompanied with habitat niche in the conventional bulk isotope method.

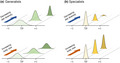

## 1 INTRODUCTION

Species can change their ecological niche (e.g., interactions among species) and function (e.g., prey‐predation, diet competition, and habitat segregation) at species and guild levels to adapt to changes in their ecological and environmental conditions. The ecological niche is a fundamental concept for understanding ecosystems and was formalized by Hutchinson (1957) as an n‐dimensional hypervolume that includes geographical diversity, resource use, and many other aspects related to the diverse interactions among species in communities and ecosystems. The n‐dimensional niche was further defined by distinguishing between “scenopoetic” and “bionomic” axes, which are frequently represented by variability in habitat and trophic niches, respectively. These axes have been used to understand the functional role of individual species in communities and ecosystems with diverse ecological, environmental, and biological conditions (Hutchinson, 1978; Newsome, Rio, Bearhop, & Phillips, 2007).

Stable isotope analysis is one of the most widely applied methods in ecological niche studies because diets and trophic hierarchies of consumers are estimated easily by stepwise enrichment of heavy isotopes (e.g., ^13^C and ^15^N) along food chains in ecosystems. Time‐integrated information, which can be readily obtained from the stable isotope analysis, is preferred to traditional approaches (e.g., gut content analysis) as the latter generally only provide a “snapshot” of a short period in the life cycle of a species (Hannides, Popp, Landry, & Graham, 2009; Rolff, 2000). The area of a polygon drawn on the cross‐plot of stable isotope ratios of two elements (e.g., carbon and nitrogen) frequently provides both “scenopoetic” and “bionomic” axes in n‐dimensional niche studies as the “δ space or isotopic niche” (Newsome et al., 2007). If variability in the isotope ratios of consumers mirrors diversity in the diet and trophic position (TP) of consumers in food webs, we can visualize the niche width of a species in an ecosystem based on the size of the δ space. For instance, Layman, Quattrochi, Peyer, and Allgeier (2007) found that ecosystem fragmentation changed the size of the δ space, resulting in niche width collapse, even within a single species. Bearhop, Adams, Waldron, Fuller, and Macleod (2004) proposed that the δ space expresses differences in the niche width size among species, being large for generalists and small for specialists. Potential competition among species in a food web can also be visualized by overlapping of the δ space among species (Hill, Jones, Hill, & Weyl, 2015).

Carbon and nitrogen isotope ratios in bulk tissues of organisms are powerful indicators of diet resources and the TP of organisms, respectively, because of small and large isotopic fractionation for carbon and nitrogen isotopes, respectively, along food chains (DeNiro & Epstein, 1981; Minagawa & Wada, 1984; Post, 2002). Therefore, niche width described with isotope ratios is defined as trophic niche width and has been employed as a bionomic axis in previous studies (Jackson, Inger, Parnell, & Bearhop, 2011; Layman & Allgeier, 2012; Navarro, Coll, Somes, & Olson, 2013). However, because the isotope ratios in primary producers, which are the base resources of food webs, often show spatial and temporal variations within a single habitat, similar to a scenopoetic axis, isotope analysis can be useful as an indicator of both trophic and habitat niches. For example, in an ecosystem where two isotopically distinct primary producers are found owing to their utilization of different inorganic nitrogen (e.g., ${\text{NO}}_{3}^{-},{\;\text{NH}}_{4}^{+}$ , and N_2_) sources, the isotope ratios of the herbivores in this ecosystem would represent not only TP, but also habitat niche (Choi et al., 2017; Newsome et al., 2007). This reflection of both trophic and habitat information, however, leads to the complexity of information provided from nitrogen isotope ratios. Therefore, to identify ecological niches correctly, the deconvolution of the trophic and habitat information is required.

Compound‐specific isotope analysis (CSIA) of nitrogen in amino acids has been recently used as a tool to estimate the TP of organisms because it allows the full separation of two isotopic changes regarding diet resources and trophic shift within a single species in ecosystems (Gangné et al., 2018; Ogawa, Chikaraishi, & Ohkouchi, 2013; Ostrom et al., 2017). This method is based on the physiological knowledge that the nitrogen isotope ratios of “source” (e.g., phenylalanine) and “trophic” (e.g., glutamic acid) amino acids have small and large isotopic fractionation, respectively, during consumer metabolism and the isotope ratios of source amino acids retain those of the diet resources whereas differences in the ratios of trophic and source amino acids correlate with the TP of the species (Chikaraishi, Kashiyama, Ogawa, Kitazato, & Ohkouchi, 2007; McCarthy, Benner, Lee, & Fogel, 2007; McClelland & Montoya, 2002; Popp et al., 2007). Applying this “CSIA of amino acids,” several previous studies have reported great accuracy in the TP estimation for both aquatic (Bowes & Thorp, 2015; Chikaraishi et al., 2014; Ishikawa, Chikaraishi, et al., 2017; Ishikawa, Hayashi, Sasaki, & Chikaraishi, 2017) and terrestrial (Chikaraishi, Ogawa, Doi, & Ohkouchi, 2011; Steffan et al., 2013, 2015) food webs. Moreover, temporal variations in the TP of consumers have been investigated regarding environmental changes, with full separation from isotopic changes on diet resources. For instance, Ogawa et al. (2013) found that the TP of the gobiid fish *Gymnogobius isaza* remained constant despite a gradual increase in the nitrogen isotope ratio of environments during a major eutrophication period in Lake Biwa through the 20th century. Temporal variations in TP were demonstrated for birds (Ostrom et al., 2017) and fish (Blanke, Chikaraishi, & Vander Zanden, 2018) in environments where the quality of diet resources had been altered by anthropogenic activities. These studies reported diverse trends of temporal variations in the TP based on accurate estimates of TP from historical samples of consumers, independent of variability in the nitrogen isotope ratios of diet resources.

Temporal changes in the TP of species potentially account for the distinct adaptabilities between generalists and specialists to ecological conditions, although the CSIA of amino acids has not been applied to compare the trophic niche width between them in ecosystems. Moreover, while previous studies successfully illustrated temporal variation in the TP (Blanke et al., 2018; Ogawa et al., 2013; Ostrom et al., 2017), spatial variation has not been demonstrated. In the present study, we investigated the variability in the TP of two different fish species: the pike gudgeon *Pseudogobio esocinus* and the largemouth bass *Micropterus salmoides*, as representative specialist and generalist species, respectively, among various ecological conditions in the four major rivers of Korea. A considerably large variation in the ecological conditions (0.0–1.8 units for the benthos diversity index and 0.4–2.4 units for the fish diversity index) among the four rivers was reported in 2017 (Ministry of Environment of Korea, 2017). Because *P. esocinus* is a benthic insectivore (Katano, Nakamura, & Abe, 2008; Katano, Nakamura, & Yamamoto, 2006), we hypothesized that the TP of *P. esocinus* in the rivers would be representative of the spatial variation of the TP of specialists in ecosystems. In contrast, *M. salmoides* is a generalist in rivers and lakes, as has been shown in previous studies of the feeding strategies of this invasive species (Dharampal & Findlay, 2017; Doi, Chang, Ando, Imai, & Nakano, 2010; Jang, Joo, & Lucas, 2006). *Micropterus salmoides* was introduced to Korea in the 1970s and spread rapidly to the four rivers during the 1990s (Jang et al., 2002). Thus, we hypothesized that the TP of *M. salmoides* among the four rivers would represent the spatial variation in the TP of generalists in ecosystems. Moreover, we expected that a comparison of the trophic niches of these two species would allow us to differentiate between the trophic functional roles of generalists and specialists in communities and ecosystems.

## 2 MATERIALS & METHODS

### 2.1 Study sites and sampling

**FIGURE 1:**
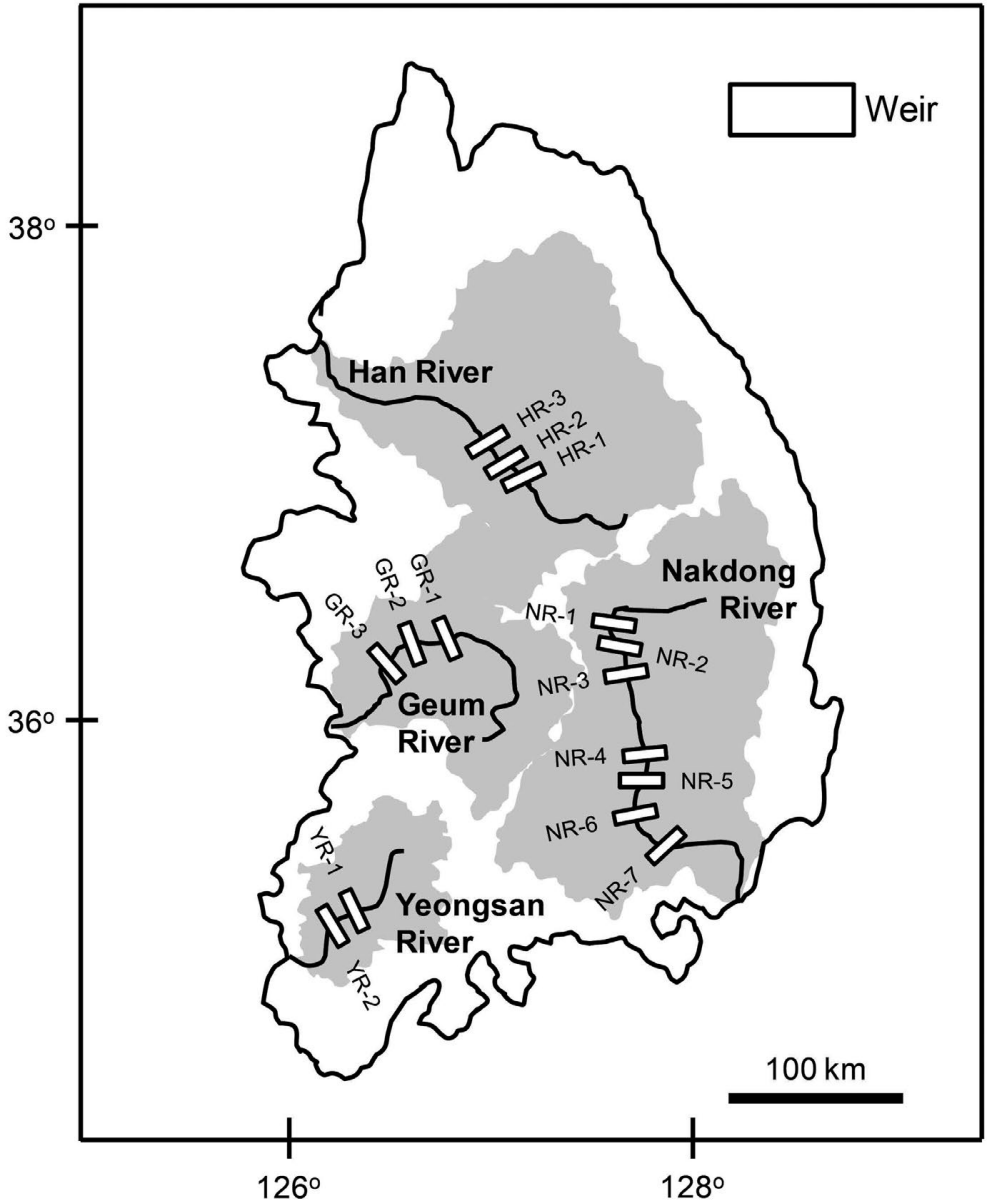
Sampling sites. Shaded area indicates the catchment area for each river and open square indicates weirs. Samples were collected upstream of each weir

Samples were collected upstream of 15 weirs in four major Korean rivers (three weirs in Han River, three weirs in Geum River, seven weirs in Nakdong River, and two weirs in Yeongsan River) in June and August 2017 (Figure 1). These weirs were constructed from 2009 to 2012 for multiple purposes including to reduce droughts and floods, improve water quality, and restore ecosystems (Hur, Jang, Shin, Lee, & Chang, 2018). The two fish species (*M. salmoides* and *P. esocinus*) were collected using three types of gear: a cast net (7 mm mesh), fixed shore nets (4 mm mesh), and skimming nets (4 mm mesh). The body lengths of the collected fish were measured and the fish were dissected to obtain the dorsal muscle tissues. Approximately 1 L of surface water was collected from each study site and filtered with a 100‐μm‐mesh filter to remove large particles and subsequently with precombusted Whatman GF/F filter paper to collect particulate organic matter (POM). Fish muscle tissues and POM were stored at −20°C until the isotope analysis.

### 2.2 Analysis of nitrogen isotope ratios of bulk tissues

Fish muscle (*n* = 5 for each site per species) and POM (*n* = 3 for each site) samples were dried in a freeze drier for 48 hr and the dried fish muscle was ground using a mortar and pestle. Isotope ratios were determined using an elemental analyzer (EA, Euro EA3028, EuroVector) coupled to an isotope ratio mass spectrometer (IRMS, Isoprime 100, Elementar). Approximately 0.8 mg of the dried sample was sealed in a tin cup and inserted into the EA and temperatures of 1,020°C and 650°C were used for the oxidation and reduction furnaces, respectively. The international isotopic reference, N−1 (0.4 ± 0.2; IAEA), was analyzed every 10 sample runs to normalize isotope ratios to the international scales (δ^15^N, ‰ vs. air). Analytical precision (1*σ* standard deviation) of the isotope analysis was better than 0.2‰.

### 2.3 Analysis of nitrogen isotope ratios of amino acids

Nitrogen isotope ratios of glutamic acid and phenylalanine in fish muscle samples of each species (*n* = 1 for each site) were determined using a gas chromatograph coupled to an IRMS (GC‐IRMS, Isoprime, Elementar) according to the procedure described by Chikaraishi et al. (2009). In brief, approximately 5 mg of the muscle tissues for each species was hydrolyzed with HCl (12 M) at 110°C for longer than 12 hr and the hydrophobic content in the hydrolysate was removed with hexane/dichloromethane (3/2, v/v). Subsequently, derivatization was performed with thionyl chloride/2‐propanol (1/4, v/v) for 2 hr at 110°C and then with pivaloyl chloride/dichloromethane (1/4, v/v) for 2 hr at 110°C. The derivatives of amino acids were extracted with *n*–hexane/dichloromethane (6/5, v/v). The δ^15^N values of the individual amino acids were determined with the GC‐IRMS system linked via a combustor (GV Instruments). An Ultra‐2 capillary column (50 m length, 0.32 mm internal diameter, 0.52 μm film thickness; Agilent Technologies) was used in the GC system with helium as the carrier gas in a constant flow mode (1.1 ml/min) and with combustion and reduction furnace temperatures of 980°C and 650°C, respectively. After every five samples run, a standard mixture of eight reference amino acids (alanine, glycine, leucine, norleucine, aspartic acid, methionine, glutamic acid, and phenylalanine) was analyzed to confirm analytical precision (1*σ* standard deviation) better than 0.8‰

### 2.4 Trophic position estimation and statistical methods

In the present study, the TP of the fish samples determined by the isotope analysis of bulk nitrogen of muscle tissues was compared with that determined by the CSIA of amino acids. Applying the bulk isotope analysis, we used the following conventional TP_bulk_ equation (Equation 1):

(1) $${\text{TP}}_{\text{bulk}}=\;\left[\left(\delta^{15}N_{\text{fish}}-\delta^{15}N_{\text{baseline}}\right)/\text{TDF}\right]+\lambda$$

where TDF was set to 3.4‰, the value established by Minagawa and Wada (1984) as the most cited value, and δ^15^N_baseline_ was assigned the δ^15^N value of POM (δ^15^N_POM_) with *λ* = 1. TP based on the CSIA of amino acids, that is, glutamic acid (δ^15^N_glu_) and phenylalanine (δ^15^N_phe_), was established using the following TP_glu/phe_ equation (Equation 2):

(2) $${\text{TP}}_{\text{glu}/\text{phe}}=\left[\left(\delta^{15}N_{\text{glu}}-\delta^{15}N_{\text{phe}}-\beta \right)/\text{TDF}\right]+1$$

where *β* was set to 3.4‰ for an aquatic food web (Chikaraishi et al., 2009) and TDF was set to 6.9‰, which was the value established by Blanke et al. (2017) based on culture experiments of freshwater fish. TP variability was determined by subtracting the average TP (TP_avg_) from the individual TP of specimens (TP_ind_) within a species as follows (Equation 3):

(3) $$\text{TP variability}={\text{TP}}_{\text{ind}}-{\text{TP}}_{\text{avg}}$$

The correlations between ecological and biological indices (e.g., fish diversity and dominance, benthic diversity index (BDI), benthic macroinvertebrate index (BMI), and body length of individual specimens) and the TP variation of each fish species were assessed using principal component analysis (PCA) and correlation analysis. The package “FactoMineR” (Lê, Josse, & Husson, 2008) was used to perform the PCA with the R program (Ver. 3.4.2) and bivariate correlation analysis with Pearson's coefficient (2‐tailed p value, *p* < .05) was performed using the SPSS Statistics software program (IBM, USA). Values of ecological indices were obtained from the report “Research on the characteristics of large river ecosystem (NIER‐SP2017‐359)” performed by the Ministry of Environment, Korea. Based on the numbers of species and their densities, a diversity index for benthos and fish was calculated using the Shannon–Wiener function (Pielou, 1966) and a dominance index was obtained using McNaughton's dominance index (McNaughton, 1967).

## 3 RESULTS

No substantial differences in the δ^15^N variation of POM, *P. esocinus*, and *M. salmoides* were observed between the two sampling seasons (June and August) (Tables 1 and 2). Spatial variation in the δ^15^N value of POM (δ^15^N_POM_) ranged from 0.1‰ to 25.6‰, which was larger than that of *P. esocinus* (from 11.6‰ to 17.4‰) and *M. salmoides* (from 11.8‰ to 20.9‰) (Table 1). The low δ^15^N_POM_ values compared to the δ^15^N values of *P. esocinus* and *M. salmoides* were consistent with general knowledge about the characteristics of basal resources and consumers in food webs (Choi et al., 2017; Ha, Won, Kim, & Shin, 2014). Based on the δ ^15^N_POM_ values, the TP_bulk_ values of *P. esocinus* (2.4 on average) were lower than those of *M. salmoides* (3.0) but with a large variation (1*σ* = 0.60 and 0.59, respectively).

**TABLE 1:** Mean value (1*σ* standard deviation) of the isotope ratios (‰) for bulk nitrogen in particulate organic matter (POM) and two fish species (*Pseudogobio esocinus* and *Micropterus salmoides*)

Name of river	Site	POM				*Pseudogobio esocinus*				*Micropterus salmoides*			
		Jun		Aug		Jun		Aug		Jun		Aug	
		Mean	1*σ*	Mean	1*σ*	Mean	1*σ*	Mean	1*σ*	Mean	1*σ*	Mean	1*σ*
Han River	HR‐1	10.2	0.9	10.2	0.9	15.7	0.3	15.0	0.6	17.4	0.6	15.7	0.7
	HR‐2	11.2	0.4	5.9	0.4	15.7	0.2	15.0	0.2	17.5	0.3	15.8	0.7
	HR‐3	11.4	0.4	15.6	0.4	‐	‐	17.3	0.5	‐	‐	18.4	0.5
Geum River	GR‐1	13.4	0.3	12.3	0.3	17.4	‐	15.4	0.5	17.8	0.3	16.1	0.7
	GR‐2	10.8	0.1	9.7	0.2	16.6	0.1	16.1	0.7	18.3	0.4	15.6	0.5
	GR‐3	9.7	0.9	10.2	0.9	‐	‐	‐	‐	18.8	‐	16.3	0.4
Nakdong River	NR‐1	9.1	2.0	5.3	0.5	15.2	0.4	‐	‐	15.8	2.7	16.9	‐
	NR‐2	12.1	1.0	6.5	0.5	16.3	‐	13.9	1.0	18.2	0.5	16.7	0.6
	NR‐3	9.3	1.6	9.2	0.2	‐	‐	‐	‐	17.6	0.7	15.8	0.6
	NR‐4	15.1	1.1	11.5	0.4	‐	‐	‐	‐	20.9	0.4	18.1	1.1
	NR‐5	11.6	1.0	12.0	0.5	‐	‐	14.7	0.2	‐	‐	18.5	0.9
	NR‐6	14.5	3.6	10.6	0.2	16.2	‐	‐	‐	20.2	0.2	18.1	0.6
	NR‐7	12.0	1.0	8.6	0.5	16.6	0.7	‐	‐	18.6	0.3	17.4	0.2
Yeongsan River	YR‐1	17.7	2.7	0.1	0.1	11.6	1.2	‐	‐	12.7	0.4	11.8	0.3
	YR‐2	16.8	1.3	7.6	0.3	‐	‐	‐	‐	13.8	0.5	‐	‐
	YR‐3^a^	10.1	1.9	25.6	0.2	‐	‐	‐	‐	16.9	0.5	16.3	0.4

^a^ YR‐3 is located downstream of the same weir as in YR‐2.

**TABLE 2:** Nitrogen isotope ratios (‰) of glutamic acid (Glu) and phenylalanine (Phe) in *Pseudogobio esocinus* and *Micropterus salmoides*

River	Site	*Pseudogobio esocinus*				*Micropterus salmoides*			
		May		Aug		May		Aug	
		Glu	Phe	Glu	Phe	Glu	Phe	Glu	Phe
Han River	HR‐1	29.4	12.4	26.0	8.2	27.3	8.4	28.2	5.9
	HR‐2	26.3	10.9	23.7	8.2	25.6	8.3	30.5	7.2
	HR‐3	‐	‐	24.5	9.0	‐	‐	34.5	12.6
Geum River	GR‐1	29.1	12.1	26.9	9.32	30.1	11.6	25.7	11.2
	GR‐2	27.6	9.3	25.7	10.2	32.2	12.1	27.5	9.0
	GR‐3	‐	‐	‐	‐	31.8	13.4	28.5	11.4
Nakdong River	NR‐1	23.9	7.3			31.4	10.5	22.0	6.9
	NR‐2	27.1	8.8	27.4	9.7	29.9	7.5	27.0	7.0
	NR‐3	‐	‐	‐	‐	27.5	7.6	25.7	8.1
	NR‐4	‐	‐	‐	‐	35.6	15.9	31.2	11.8
	NR‐5	‐	‐	27.7	10.8	‐	‐	26.5	10.3
	NR‐6	24.9	8.6	‐	‐	32.1	12.1	25.0	8.6
	NR‐7	27.8	10.1	‐	‐	29.4	9.7	27.0	11.1
Yeongsan River	YR‐1	22.4	5.0	‐	‐	24.1	1.6	27.3	7.3
	YR‐2	‐	‐	‐	‐	29.3	6.7	‐	‐
	YR‐3^a^	‐	‐	‐	‐	32.6	8.0	30.8	10.2

^a^ YR‐3 is located downstream of the same weir as in YR‐2.

The δ^15^N values of glutamic acid (δ^15^N_glu_), a representative trophic amino acid, in both *P. esocinus* (from 22.4‰ to 29.4‰) and *M. salmoides* (from 22.0‰ to 35.6‰) were higher than those of phenylalanine (δ^15^N_phe_), a representative source amino acid, in *P. esocinus* (from 5.0‰ to 12.4‰) and *M. salmoides* (from 1.6‰ to 15.9‰) (Table 2). This δ^15^N trend between glutamic acid and phenylalanine was consistent with the general trend reported in previous studies (Chikaraishi et al., 2007; McCarthy et al., 2007; McClelland & Montoya, 2002; Popp et al., 2007). The δ^15^N_glu_ values in *M. salmoides* (28.8 ± 3.2‰) were larger than those in *P. esocinus* (26.3 ± 2.0‰), whereas the δ^15^N_phe_ values were almost identical between *P. esocinus* (9.4 ± 1.8‰) and *M. salmoides* (9.4 ± 2.8‰). The slightly higher TP_glu/phe_ values in *M. salmoides* (3.3 ± 0.3) than that in *P. esocinus* (3.0 ± 0.1) were similar to the TP_bulk_ values in these species (3.0 and 2.4, respectively); however, the TP_glu/phe_ values were higher than the TP_bulk_ values.

**FIGURE 2:**
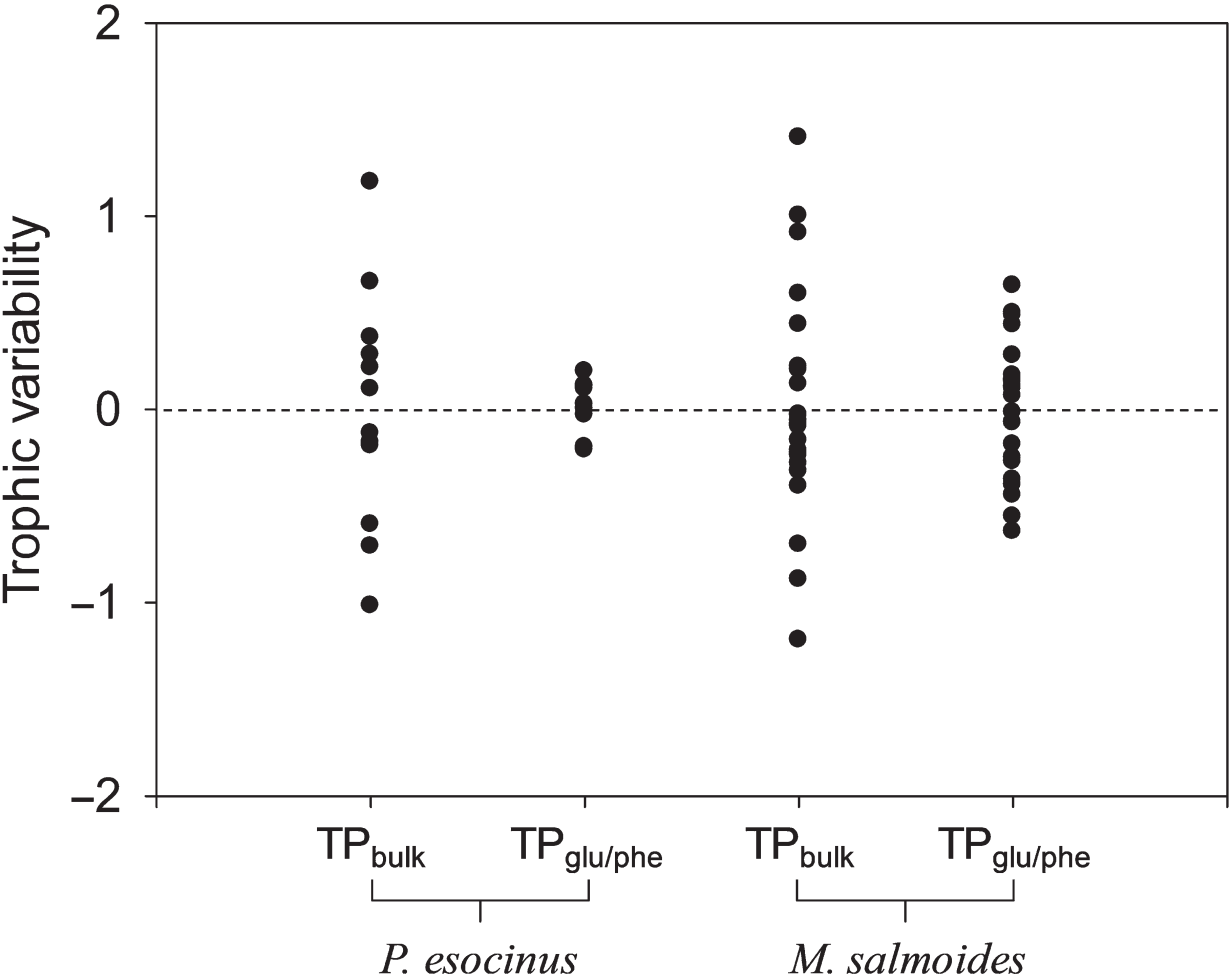
Trophic variability of *Pseudogobio esocinus* and *Micropterus salmoides* estimated by the bulk isotope analysis and CSIA of amino acids

To evaluate TP variability, we performed normalization using Equation (3). The range of variability in the TP_glu/phe_ value (from −0.2 to 0.2) was much smaller than that in the TP_bulk_ value (from −0.9 to 1.2) in *P. esocinus* (Figure 2). Considering the analytical errors in the TP_glu/phe_ value (1*σ* < 0.2), *P. esocinus* had remarkably uniform TP_glu/phe_ values across sampling sites. In contrast, the range of variability in the TP_glu/phe_ value (from −0.6 to 0.6) in *M. salmoides*, although smaller than that in the TP_bulk_ value (from −1.2 to 1.4), was still much larger than that in *P. esocinus*, indicating significant TP variability in *M. salmoides* based on TP_glu/phe_.

**FIGURE 3:**
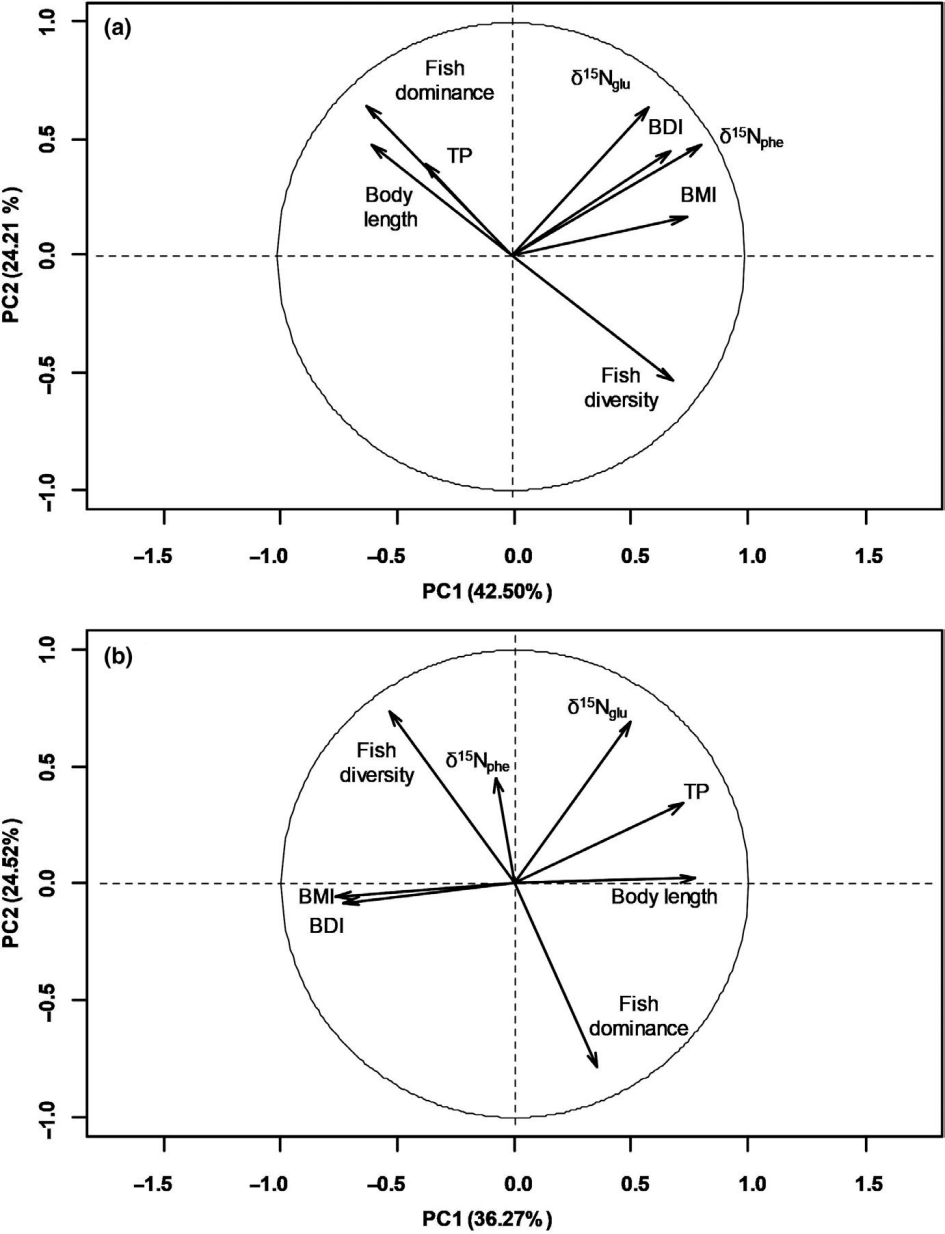
Principal component analysis associating ecological and biological indices with TP and nitrogen isotope ratios of amino acids in (a) *Pseudogobio esocinus* and (b) *Micropterus salmoides*

The PCA demonstrated that the TP variability of *P. esocinus* had a positive relationship with fish dominance and body length, a negative relationship with fish diversity, and no relationship with benthos indices (Figure 3a). However, these positive and negative relationships were not significant based on the bivariate correlation analysis results (*p* > .5, Table 3). In contrast, the TP variability of *M. salmoides* had a positive relationship with body length and a negative relationship with benthic indices (Figure 3b, *p* < .05 for both); however, it had no relationship with fish diversity or dominance (bivariate correlation analysis, *p* > .1, Table 3).

**TABLE 3:** Bivariate correlation analysis results and Pearson's coefficients of the relationships between the trophic positions of two fish species (*Pseudogobio esocinus* and *Micropterus salmoides*) and various ecological and biological indices

Parameter	Pearson's correlation	*p* value
*Pseudogobio esocinu*s (*n* = 16)		
BMI	−0.355	.177
BDI	−0.239	.373
Body length	0.471	.076
Fish diversity	−0.219	.415
Fish dominance	0.289	.277
δ^15^N_glu_	0.456	.076
δ^15^N_phe_	−0.043	.873
*Micropterus salmoides* (*n *= 20)		
BMI	−**0.445** *	**.016**
BDI	−**0.516** **	**.004**
Body length	**0.458** *	**.012**
Fish diversity	−0.115	.554
Fish dominance	−0.134	.489
δ^15^N_glu_	**0.533** **	**.003**
δ^15^N_phe_	−0.306	.107

* Correlation is significant at the 0.05 level (2‐tailed).

** Correlation is significant at the 0.01 level (2‐tailed).

## 4 DISCUSSION

Enrichment in ^15^N along food chains has been used widely to estimate the consumer TP for several decades. In particular, the CSIA of amino acids can provide trophic information regarding consumers, independent of variation in the δ^15^N value of diet resources, resulting in highly accurate TP estimates (Chikaraishi et al., 2014; Hannides et al., 2009; Sackett, Drazen, Choy, Popp, & Pitz, 2015). The variability of TP_glu/phe_ was noticeably smaller than that of TP_bulk_ in *P. esocinus* in the present study (Figure 1). The large variability in the TP_bulk_ of *P. esocinus* was because the TP_bulk_ reflects not only trophic elevation but also spatial and temporal variations in the δ^15^N value of the diet resources of *P. esocinus*, which was shown by the spatial variation in the δ^15^N value of POM (5.9‰ to 17.7‰, Table 1). In contrast, the small variability in the TP_glu/phe_ value of *P. esocinus* indicated that the TP information (TP_glu/phe_) was successfully isolated from the spatial and temporal variations in the δ^15^N value of diet resources. Thus, eliminating the influence of δ^15^N variations in the diet resource, we showed that *P. esocinus* preferred stable, trophically uniform diets even in different habitats, regardless of body size. In contrast, the variability of TP_glu/phe_ in *M. salmoides* was larger than that in *P. esocinus*, which indicated that *M. salmoides* can change diets based on habitats, body size, or both. In previous studies, the trophic niche width estimated using the bulk isotope analysis generally distinguished well between specialist and generalist species (Bearhop et al., 2004; Vander Zanden, Bjomdal, Reich, & Bolten, 2010). Highly selective feeding preferences of specialists are expressed by a narrow δ space (i.e., trophic niche width) with nearly identical isotope ratios among multiple specimens for a single species (Vander Zanden et al., 2010), whereas the flexible feeding strategies of generalists are expressed by a wide δ space with variable isotope ratios among multiple specimens (Layman & Allgeier, 2012; Sanders, Vogel, & Knop, 2015). Thus, the small and large variabilities in the TP_glu/phe_ values for *P. esocinus* and *M. salmoides*, respectively, were highly correlated with the expected trophic niche widths for specialists and generalists, respectively.

The PCA results for *M. salmoides* further revealed that variability in TP was negatively correlated with BMI and BDI, and positively correlated with body size (Figure 3b). These negative correlations imply that the diversity of benthic species is a major factor controlling the TP of *M. salmoides*, which is consistent with the results of a previous study (Doi et al., 2010) where the contribution of benthic invertebrates to the diet of *M. salmoides* varied significantly among habitats with different levels of human activities. Moreover, another study reported that the integrated TP of the benthos was decreased with an increase in benthic diversity of stream ecosystems (Ishikawa, Chikaraishi, et al., 2017; Ishikawa, Hayashi, et al., 2017). Thus, the negative correlation between benthic indices and the TP of *M. salmoides* in the present study can be explained by benthic invertebrates as a primary factor. The high TP of *M. salmoides* may be caused by the consumption of high TP diets (e.g., fish) when the benthic community is limited. TP variations in *M. salmoides* and other generalist carnivores (e.g., seabirds) have often been interpreted by changes in the proportions of diets because the TP change in seabirds over historical time scales related to the change in the diet proportion due to overfishing by humans (Becker & Beissinger, 2006; Ostrom et al., 2017).

The positive correlation between body size and TP variability in *M. salmoides* demonstrated that body size was also an important factor affecting the TP of this species. Unlike the benthos indices (i.e., BMI and BDI), which mirror differences in the habitat property, body size index mirrors differences in the diet preference between specimens within this species. An ontogenetic variation in the diet preference of generalists has been observed in several species (Estes, Riedman, Staedler, Tinker, & Lyon, 2003; Kim, Tinker, Estes, & Koch, 2012; Post, 2003). Diet shifts in piscivores are likely dependent on the balance between the availability of dietary items and body size that is adequate to consume these items (Mittelbach & Persson, 1998; Olson, 1996). Post (2003) demonstrated that the timing of a diet shift in *M. salmoides* was individually variable during the young stages. Therefore, the positive correlation between body size and TP (Figure 3 and Table 3) indicates that these two factors (diet shift and different habitat) affected the TP when fish were small, but only habitat characteristics were important when predatory fish were large. Fish diversity and dominance have no significant correlation to TP variability in *M. salmoides*, even though other generalist fish potentially play roles in diets and competitors (Jang et al., 2006; Raborn, Miranda, & Driscoll, 2004). Thus, our results indicate that (a) two diet indices (i.e., BMI and BDI) of benthic communities in the habitat of *M. salmoides* and (b) the ontogenesis of *M. salmoides* affect the trophic niche width of this generalist species rather than fish diversity and dominance as potential indicators for diet competition and fish diet in ecosystems.

In contrast to *M. salmoides*, negligible variation was apparent in the TP of *P. esocinus* in the present study (Figure 2), which was consistent with there being no significant correlation of the TP variability for this species with indices of BMI, BDI, and body size, as well as fish diversity and dominance (Figure 3 and Table 3). The negligible TP variability of *P. esocinus* implies that *P. esocinus* has a trophically uniform diet (e.g., aquatic insects; Katano et al., 2008) even in different habitats and at different body sizes. The trophic niche of *P. esocinus* demonstrated in the present study was consistent with the general concept of the specialist being at high risk of extinction owing to their lack of ability to shift their diets (Layman et al., 2007). Considering its wide distributed population to almost all rivers and lakes in Korea, as well as Japan and northern China (Tominaga, Nakajima, & Watanabe, 2016), the absence of *P. esocinus* at several sites in the present study might be related to the restriction of habitats for this species. Analysis of the variation in the TP of consumers and its resulting trophic niche width can thus be useful for evaluating the effects of natural and artificial changes on ecosystems. The present study demonstrated that the CSIA of amino acids allowed the accurate estimation of the TP dynamics of consumers independent of spatial and temporal variations in the δ^15^N value of diet resources. Moreover, subsequent statistical analysis with related indices allowed us to estimate how individual ecological and biological factors influence the trophic variability of specialists and generalists within given habitats.

**FIGURE 4:**
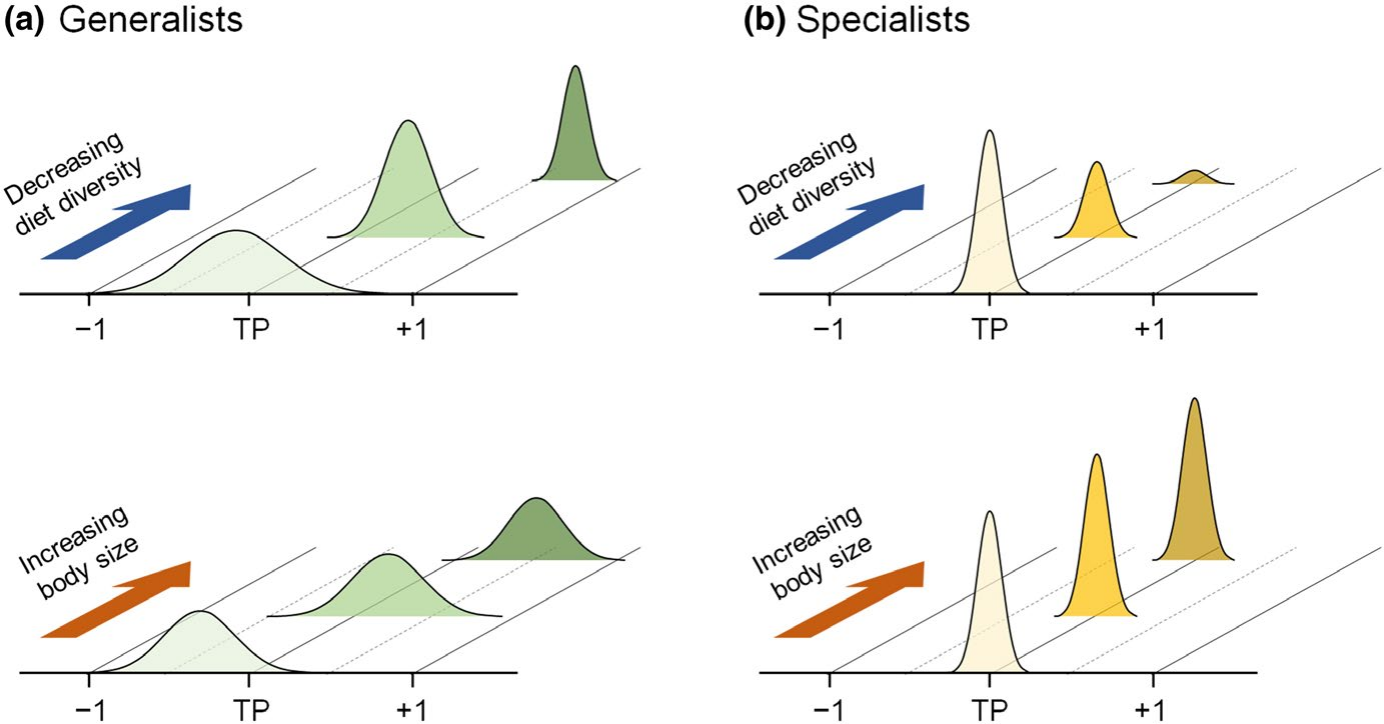
Schematic of trophic variability in (a) generalists and (b) specialists controlled by diet diversity and body length based on the present study

The trophic niche of consumers has been widely used to evaluate the influence of human activities on food webs (Layman et al., 2007; Ogawa et al., 2013; Ostrom et al., 2017; Park et al., 2019) and these types of studies have been strengthened considerably by the CSIA of amino acids that can detect slight changes in the trophic niche of historical samples (Ogawa et al., 2013; Ostrom et al., 2017). Several studies have reported no substantial change in TP of consumers despite environmental changes (Chikaraishi et al., 2014; Hannides et al., 2009; Ogawa et al., 2013) and these results have been extrapolated to the ecosystem based on the assumption that the consumers are representative species in the studied ecosystems. However, our results in the present study highlight the importance of the careful selection of target consumers, particularly for either generalists or specialists, to assess correctly the effects of ecosystem changes. Based on the present study, the TP of generalists shifts with diet diversity, implying that the TP of generalists can be useful for evaluating trophic responses to ecological conditions, although further consideration of TP variation with body size is required (Figure 4a). In contrast, for specialists, TP barely shifts with diet diversity and body size whereas the population is potentially controlled by diet diversity, implying that the population of specialists can be useful for evaluating environmental changes in response to human activities (Figure 4b).

**FIGURE 5:**
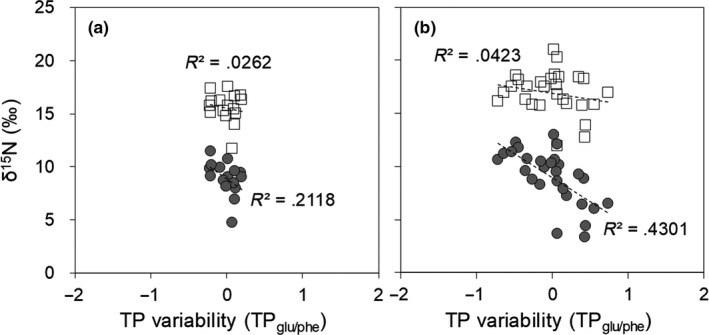
Relationship of trophic variability with nitrogen isotope ratios of bulk tissues (open square) and estimated nitrogen isotope ratios of potential diet resources (gray circle) in (a) *Pseudogobio esocinus* and (b) *Micropterus salmoides*

Trophic and diet resource information coexists in the δ^15^N value of bulk tissues, and therefore, the uncertainty of trophic information is frequently large if the δ^15^N values are used solely as a trophic indicator. Our findings for the poor correlation between the TP_glu/phe_ and δ^15^N values in bulk tissues of *M. salmoides* demonstrate the risk in the use of bulk isotope analysis (Figure 5b). However, even in the case of little variation in the TP_glu/phe_, the δ^15^N values of bulk tissues for *P. esocinus* had a large variation (11‰ to 17‰), implying that the δ^15^N values of diet resources had a large spatial variation (Table 1 and Figure 5a). We can extract spatial differences in the habitat if trophic information is eliminated from the δ^15^N values of bulk tissues. Such bulk δ^15^N values, which contain information of longer time integration than that of POM, can be simply calculated using Equation (1) with the TP_glu/phe_ of each fish species. The estimated δ^15^N values of the potential diet resources for *P. esocinus* were considerably lower than the original δ^15^N values of this fish and remained a large spatial and temporal variation with no (or very weak) correlation with the TP_glu/phe_ variability (Figure 5a). In contract, for *M. salmoides*, the estimated δ^15^N values of the potential diet resources had a negative correlation with TP_glu/phe_ variability (*R* ^2^ = 0.4031), whereas the original δ^15^N values of this fish had substantially no correlation with the TP (Figure 5b). This negative correlation for *M. salmoides* supports our hypothesis that the TP of *M. salmoides* decreased with a higher contribution of benthos diets (which are derived from benthic resources) than fish diets (which are derived from pelagic resources), considering that the former has higher δ^15^N values than the latter in the present study.

The dual plot for the TP information obtained by CSIA of amino acids and estimated δ^15^N value of potential diet resources can be useful for evaluating the ecological niche of individual generalist fish species. This approach allowed us to have a simple separation between habitat (diet) and trophic niche information in the δ space, unlike traditional bulk isotope analysis that cannot separate this information in the space.

## 5 CONCLUSION

The increasing effects of human activities on natural ecosystems are one of the most important issues in ecology. In the present study, we applied the CSIA of amino acids to understand the trophic variability of a specialist and generalist fish species in ecologically variable habitats on different river ecosystems. We found distinct correlations for the TP_glu/phe_ of specialists and generalists with ecological indices such as BDI and BMI, indicating different adaptation strategies between these species to ecological conditions. Because numerous environmental and biological factors can affect ecological conditions and frequently multiple factors interact with each other, it is still unknown whether our finding of a relationship between TP and diet diversity applies to other ecosystems. However, we predict that this CSIA approach will be useful as a powerful tool to evaluate the relationship among TP and the ecological conditions between functional species (e.g., specialists vs. generalists, benthic vs. pelagic, and herbivore vs. carnivore) for diverse ecosystems.

## CONFLICT OF INTEREST

None declared.

## AUTHOR CONTRIBUTIONS

**Bohyung Choi:** Conceptualization (equal); Formal analysis (equal); Methodology (equal); Visualization (equal); Writing‐original draft (equal); Writing‐review & editing (equal). **Changhwa Lee:** Conceptualization (equal); Formal analysis (equal); Writing‐original draft (supporting). **Yuko Takizawa:** Visualization (equal); Writing‐original draft (equal); Writing‐review & editing (equal). **Yoshito Chikaraishi:** Visualization (equal); Writing‐original draft (equal); Writing‐review & editing (equal). **Hye‐Ji Oh:** Formal analysis (equal); Investigation (equal). **Kwang‐Hyeon Chang:** Conceptualization (equal); Formal analysis (equal); Investigation (equal); Methodology (equal). **Min‐Ho Jang:** Conceptualization (equal); Formal analysis (equal); Funding acquisition (equal); Investigation (equal). **Hyun‐Woo Kim:** Conceptualization (equal); Formal analysis (equal); Investigation (equal). **Kyung‐Lak Lee:** Conceptualization (equal); Funding acquisition (equal); Project administration (equal). **Kyung‐Hoon Shin:** Conceptualization (equal); Data curation (equal); Validation (equal); Writing‐original draft (lead); Writing‐review & editing (lead).

## Data Availability

δ^15^N values of bulk tissue and amino acids of samples: Dryad https://doi.org/10.5061/dryad.v15dv41s7.
